# Are cancer-related decision aids appropriate for socially disadvantaged patients? A systematic review of US randomized controlled trials

**DOI:** 10.1186/s12911-016-0303-6

**Published:** 2016-06-06

**Authors:** Kimberly R. Enard, Patricia Dolan Mullen, Geetanjali R. Kamath, Nickell M. Dixon, Robert J. Volk

**Affiliations:** Department of Health Management and Policy, Saint Louis University, 3545 Lafayette Avenue, Saint Louis, MO USA; Department of Health Promotion and Behavioral Sciences, The University of Texas School of Public Health, 7000 Fannin Street, UCT Suite 2522, Houston, TX 77030 USA; Department of Health Services Research, The University of Texas MD Anderson Cancer Center, P.O. Box 301402, Unit 1444, Houston, TX USA; Michigan Department of Health and Human Services, 201 Townsend Street, Lansing, MI 48913 USA

**Keywords:** Cancer, Decision aids, Decision-making, Disparities, Social disadvantage

## Abstract

**Background:**

Shared decision-making (SDM) is considered a key component of high quality cancer care and may be supported by patient decision aids (PtDAs). Many patients, however, face multiple social disadvantages that may influence their ability to fully participate in SDM or to use PtDAs; additionally, these social disadvantages are among the determinants of health associated with greater cancer risk, unwarranted variations in care and worse outcomes. The purpose of this systematic review is to describe the extent to which disadvantaged social groups in the United States (US) have been included in trials of cancer-related PtDAs and to highlight strategies, lessons learned and future opportunities for developing and evaluating PtDAs that are appropriate for disadvantaged populations.

**Methods:**

We selected cancer-related US studies from the Cochrane 2014 review of PtDAs and added RCTs meeting Cochrane criteria from searches of PubMed, CINAHL, PsycINFO (January 2010 to December 2013); and reference lists. Two reviewers independently screened titles/abstracts; three reviewers independently screened full text articles, performed data extraction and assessed: 1) inclusion of participants based on seven indicators of social disadvantage (limited education; female gender; uninsured or Medicaid status; non-U.S. nativity; non-White race or Hispanic ethnicity; limited English proficiency; low-literacy), and 2) attention to social disadvantage in the development or evaluation of PtDAs.

**Results:**

Twenty-three of 39 eligible RCTs included participants from at least one disadvantaged subgroup, most frequently racial/ethnic minorities or individuals with limited education and/or low-literacy. Seventeen studies discussed strategies and lessons learned in attending to the needs of disadvantaged social groups in PtDA development; 14 studies targeted disadvantaged groups or addressed subgroup differences in PtDA evaluation.

**Conclusions:**

The diversity of the US population is represented in a majority of cancer-related PtDA RCTs, but fewer studies have tailored PtDAs to address the multiple social disadvantages that may impact patients’ participation in SDM. More detailed attention to the comprehensive range of social factors that determine cancer risk, variations in care and outcomes is needed in the development and evaluation of PtDAs for disadvantaged populations.

**Trial registration:**

Registered 24 October 2014 in PROSPERO International prospective register of systematic reviews (CRD42014014470).

**Electronic supplementary material:**

The online version of this article (doi:10.1186/s12911-016-0303-6) contains supplementary material, which is available to authorized users.

## Background

The social determinants of health (SDH) are conditions in which people live, learn, work and play that interact with individual-level characteristics (e.g., age, gender, genetics, behavior) to affect a wide range of health risks and outcomes [1–3]. In the cancer care continuum, rates of incidence and death for the most common cancer types vary considerably within and across socioeconomic groups [4]. However, patients in the United States (US) who are members of certain disadvantaged subgroups (e.g., racial/ethnic minorities, the uninsured or underinsured and individuals with limited education, low income or unhealthy living conditions) are more likely than those in advantaged groups to be diagnosed with cancer at later stages [4–9], undergo greater variation in screenings and treatments received [4, 5, 8–11], and experience higher rates of morbidity and mortality [4, 5, 8, 9, 12, 13]. Compared to those in advantaged groups, cancer patients in disadvantaged subgroups are also more likely to report worse patient-provider communication and quality of care [14].

In recent years, the call to address SDH as key drivers of health inequities has gained momentum among health system leaders and policymakers in the US. Objectives outlined in Healthy People 2020 emphasize the importance of addressing SDH as one of four overarching goals for the decade [2]. New research also identifies the need to move beyond simply recognizing the fundamental role upstream SDH play in influencing health toward using this knowledge to develop and evaluate interventions that examine the mechanisms through which SDH influence downstream processes of care and outcomes [1, 15–18]. Interventions involving patient decision aids (PtDAs), developed with attention to SDH, represent important opportunities to influence patient-provider communication and shared decision making (SDM) processes.

PtDAs are tools (e.g., brochures, videos, internet-based programs) designed to help individuals participate in decisions about their healthcare by preparing them to discuss and make informed, values-based decisions in partnership with their providers [19–21]. The Institute of Medicine (IOM) has highlighted the need to support engagement in SDM, using PtDAs when available, as integral to delivering high quality cancer care [22]. In order to accomplish this goal, PtDAs must be appropriate for their intended audiences and present factual, balanced information in easy-to-understand formats that help patients consider the pros and cons of the options available to them. Consensus-based standards offer guidance on what should be included in the PtDAs and how the tools should be developed, including detailed instructions about using plain language to support the needs of people with limited reading skills [23, 24]. The standards, however, are largely silent about addressing the needs of people who face other social disadvantages that may influence health care decision making [1, 25, 26].

The multiple causal pathways through which social factors are theorized to shape health are long and complex [1, 17, 18, 27, 28]. We posit that SDH may influence patient-provider interactions and SDM by limiting the actual and/or perceived options available to patients. For example, limited education, literacy or English proficiency (LEP) may obstruct patient-provider communication, even when translators are available, or limit patients’ perceived ability to act on the information they receive [29–31]. Economic instability (e.g., low income, hourly employment, transient housing) may cause patients to delay or forego needed care because they are unable to afford co-pays/cost-shares, to purchase or appropriately store prescribed therapies, or to take time away from work for longer-term treatments [1, 17]. It may also limit one’s ability or willingness to use interventions, such as PtDAs, that require reliable computer and/or high speed internet access [32, 33]. Limitations of the built environment (e.g. inadequate access to healthy foods, transportation, safe parks) may cause patients not to choose treatment options that require lifestyle and behavior changes or travel to certain areas for treatment [1, 17]. Lack of family/social support may render home- and/or community-based treatments less desirable, while social norms/attitudes (e.g., perceived and/or actual discrimination) may lead patients and their families to distrust the advice of their providers or health system officials [1, 17, 34, 35]. Most certainly, access to healthcare (e.g., access to providers, insurance status) may reduce patients’ actual or perceived access to certain providers/facilities and/or specific treatments not covered by their insurance plans [2, 25, 27, 34]. In other words, when presented with more than one option, patients may not perceive that the full range of options is available to them based on their personal circumstances. We argue that PtDAs for disadvantaged patients should be tailored to address SDH because such tools can then help them think through and, importantly, share with their clinicians concerns about how SDH-related barriers influence their preferences for treatment.

Despite the increasing number of PtDAs being developed, little is known about the availability of cancer-related PtDAs evaluated in the US that address the pathways through which SDH may influence the decision making processes of disadvantaged patients. The purpose of this review is to address this gap in the literature. We specifically aim to answer the questions: 1) To what extent have disadvantaged social groups been included in randomized controlled trials (RCTs) of cancer-related PtDAs conducted in the US; and 2) What are the strategies and lessons learned about developing and evaluating PtDAs that address one or more SDH or otherwise support the decision making needs of disadvantaged audiences?

## Methods

### Eligibility criteria, search methods, study selection and data extraction

We examined all RCTs of PtDAs that were found in the 2014 updated Cochrane systematic review of patient decision aids [20] plus 6 other trials identified in an independent search of PubMed, CINAHL and PsycINFO (January 2010 through December 2013) using terms similar to the Cochrane protocol (Additional file 1). We included studies that met the Cochrane criteria; that is, they used a RCT design and evaluated a PtDA as part of the intervention. We eliminated duplicate studies and excluded studies that were not conducted in the US, did not target cancer-related decisions, or did not focus on active decision making. We focus our review on the US because there are major differences between the US and other high income countries in the way healthcare is financed and delivered that may impact patient-provider interactions and SDM processes. These differences are particularly relevant when examining socially disadvantaged patients. Neither study quality nor the neutrality of the PtDA was considered in judging eligibility for inclusion; those assessments are available elsewhere for studies included in the Cochrane review [20]. Reference lists from the RCTs were also searched (and references of those references, if necessary) to identify related articles reporting quantitative and/or qualitative information relevant to development of the PtDAs. Two authors (KRE, GRK) independently reviewed article titles/abstracts to assess eligibility based on our inclusion/exclusion criteria. Three authors (KRE, GRK, NMD) independently reviewed the full text of potentially eligible articles and extracted specified data using an electronic form, and disagreements were resolved by consensus; KRE aggregated the results. When available, multiple reports from the same study were reviewed for relevant data but counted as a single study.

### Measures

#### Inclusion of disadvantaged subgroups in RCTs

We evaluated inclusion of disadvantaged subgroups in the selected RCTs based on documentation of seven sample characteristics related to social disadvantage: limited education; female gender; uninsured or Medicaid status; non-US nativity; non-White race or Hispanic ethnicity; limited English proficiency (LEP); and low-literacy. These population characteristics are associated with inequities in healthcare access and, because they are recommended in reporting guidelines for systematic reviews focused on health equity [36], they were expected to be readily available in published studies. For disadvantaged subgroups other than those with LEP or low-literacy, we classified studies as inclusive when a proportion of the sample met or exceeded US national averages for the specified subgroup [37, 38]. Studies in which individuals with LEP were eligible to participate and studies that targeted low-literacy populations were also considered inclusive. Subgroup definitions for each criterion are given here:

1. **Limited Education:** ≥ 13 % less than high school (HS), ≥ 30 % HS or general educational development (GED) certificate, cumulatively, ≥ 43 % HS or less;
2. **Female Gender**: ≥ 50 % female, unless gender-specific condition;
3. **Uninsured or Medicaid Status**: ≥ 16 % uninsured, ≥ 16 % Medicaid beneficiaries or, cumulatively, ≥ 32 % uninsured or Medicaid beneficiaries;
4. **Non-US Nativity**: ≥ 12 % born outside the US;
5. **Non-White Race or Hispanic Ethnicity**: ≥ 13 % Black, ≥ 16 % Hispanic, ≥ 5 % Asian/Native Hawaiian or Pacific Islander (Asian/NHPI) or < 63 % non-Hispanic White;
6. **LEP:** study facilitated participation using ≥ 1 language other than English;
7. **Low-Literacy**: study measured and reported participants’ literacy or health literacy as low, or described participants as being from low-literacy populations.

We coded studies as “Yes” (met a criterion), “No” (did not meet a criterion or did not report data related to a criterion) or “Not Applicable” (criterion was not applicable to the cancer context; e.g., breast cancer studies limited to female patients; prostate cancer studies limited to male patients).

### Attention to SDH in PtDA development

To address this aim, we examined the included RCTs and related articles that we were able to identify through cited and citing references for documentation of a systematic process that explicitly investigated the needs of one or more disadvantaged subgroups in the development of the PtDA. We distinguish this from the previous measure (inclusion) because an RCT may have included disadvantaged groups in the samples but provided no evidence of attention to social disadvantage in how the aid was developed. We also reviewed sources for study context; eligibility criteria; identification of a conceptual framework; descriptions of the PtDA format; intended delivery mode/setting/timing; subgroups targeted in the development process; and development strategies used to inform PtDA content and to test the usability/feasibility of the intervention. We defined usability/feasibility testing as activities intended to assess participants’ ability to understand and complete intervention-related tasks; time required to complete such tasks; satisfaction with (acceptability) and estimated or actual use of the intervention (demand); and other activities related to the assessment of intervention feasibility [37].

### Attention to SDH in PtDA evaluation

We examined articles for any discussion of the effectiveness of the PtDA in a disadvantaged subgroup based on: 1) the authors’ stated *a priori* objectives to evaluate the effectiveness of the PtDA in the targeted subgroup(s), 2) use of evaluation tools specifically designed for disadvantaged subgroups (e.g., low-literacy version of a validated questionnaire), or 3) stratified subanalyses and/or PtDA-by-subgroup interactions. Here, we distinguish attention to SDH in PtDA evaluation from our measure of inclusion because studies may have failed to meet our criteria for including socially disadvantaged groups in the sample but still considered social disadvantage in the analytic plan. In contrast, studies may have met our criteria for including disadvantaged groups in the samples but failed to consider subgroup differences in the analytic plan. In the latter case, inferences about disadvantaged groups cannot be made.

## Results

### Characteristics of the RCTs

**Fig. 1:**
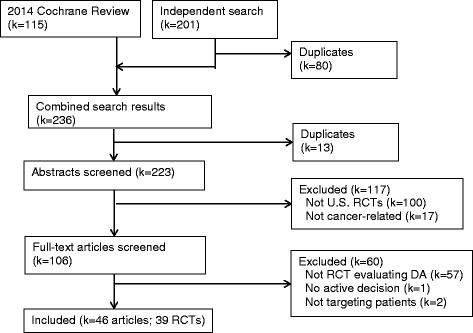
Flow Diagram of Study Selection

The initial literature search returned 223 unique references; 106 were selected for full-text review (Fig. 1). Of these references, 39 RCTs of PtDAs reported in 46 articles [38–83] published between 1995 and 2013 met our inclusion criteria (Table 1). The RCTs addressed three cancers: breast (BCa, *k* = 12), colorectal (CRC, *k* = 7) and prostate (PCa, *k* = 20); and primarily supported screening or prevention decisions (*k* = 32). The BCa studies focused on genetic testing/counseling (*k* = 5), high risk prevention (*k* = 3), reconstruction surgery (*k* = 1), and breast conserving therapy (BCT) versus mastectomy (*k* = 3). Six CRC studies addressed screening (fecal occult blood testing, flexible sigmoidoscopy, colonoscopy, double contrast barium enema); one addressed microsatellite instability (MSI) genetic testing. Seventeen studies evaluated PtDAs designed to support PCa screening decisions (prostate specific antigen testing or digital rectal exam); and three studies addressed PCa treatment (active surveillance, prostatectomy, radiation therapy or medication). Nearly all studies were conducted in urban/suburban areas; only two studies were conducted in rural areas. Regional variation was relatively evenly distributed.

**Table 1:** Inclusion of Disadvantaged Social Groups in RCTs of Cancer-Related PtDAs, by Cancer Context (*k* = 39)

			Met/exceeded US average:					Limited english proficiency	Low-literacy
Study	Context	Location (*n*)	≤HS/GED	Female	Uninsured or medicaid	Non US Nativity	Non-white or Hispanic		
BCa, Genetic Testing (*k* = 5)									
Green 2001 [38]	BRCA testing	Urban-South (*n* = 72)	No	NA	No	No	No	No	No
Green 2004 [39]	BRCA testing	Urban-MW/NE/South (*n* = 211)	No	NA	No	No	No	No	No
Lerman 1997 [40]	BRCA testing	Urban-South (*n* = 400)	No	NA	No	No	Yes	No	No
Miller 2005 [41]	BRCA testing	National (*n* = 279)	No	NA	No	No	No	No	No
Schwartz 2001 [42]	BRCA testing	Urban-South (*n* = 381)	No	NA	No	No	No	No	No
BCa, High Risk Prevention (*k* = 3)									
Fagerlin 2011 [43–45]	Chemoprevention	Urban-MW/West (*n* = 1,197)	No	NA	No	No	No	No	No
Ozanne 2007 [46]	Lifestyle v. chemoprevention v. surgery	Urban-West (*n* = 30)	No	NA	No	No	No	No	No
Schwartz 2009 [47, 48]	Mastectomy for BRCA1/2 carriers	Urban-NE/South (*n* = 214)	No	NA	No	No	No	No	No
BCa, Treatment (*k* = 4)									
Heller 2008 [49]	Breast reconstruction	Urban-South (*n* = 133)	No	NA	No	No	No	No	No
Jibaja-Weiss 2011 [50]	BCT v. mastectomy	Urban-South (*n* = 76)	No	NA	Yes	No	Yes	Yes	Yes
Marcus 2013 [51]	BCT v. mastectomy	National (*n* = 340)	No	NA	No	No	Yes	No	No
Street 1995 [52]	BCT v. mastectomy	Urban-South (*n* = 60)	Yes	NA	No	No	No	No	No
CRC, Screening (*k* = 6)									
Dolan 2002 [53]	FOBT/FS/ COL/ DCBE	Urban-NE (*n* = 95)	Yes	Yes	No	No	No	No	No
Miller 2011 [56]	FOBT/FS/COL	Urban-South (*n* = 264)	Yes	Yes	Yes	No	Yes	No	Yes
Pignone 2000 [57]	FOBT/FS	Urban-South (*n* = 249)	Yes	Yes	No	No	Yes	No	No
Ruffin IV 2007 [58]	FOBT/FS/ COL	Urban/Rural-MW (*n* = 174)	No	Yes	No	No	Yes	No	No
Schroy 2011 [59, 60]	FOBT/FS/COL/ DCBE	Urban-NE (*n* = 825)	Yes	Yes	Yes	No	Yes	No	No
Wolf 2000 [61]	FOBT/FS	Urban-NE/South (*n* = 402)	Yes	Yes	No	No	No	No	No
CRC, Genetic Testing (*k* = 1)									
Manne 2010 [54, 55]	MSI testing	Urban-NE/South (*n* = 213)	No	No	No	No	No	No	No
PCa Screening (*k* = 17)									
Allen 2010 [62]	PSA testing	Urban-NE (*n* = 812)	No	NA	No	No	No	No	No
Chan 2011 [63]	PSA testing	Urban-South (*n* = 317)	Yes	NA	Yes	Yes	Yes	Yes	No^a^
Frosch 2003 [64]	PSA testing	Urban-West (*n* = 226)	No	NA	No	No	No	No	No
Frosch 2008 [65]	PSA testing	Urban-West (*n* = 611)	No	NA	No	No	No	No	No
Krist 2007 [66]	PSA testing	Urban-South (*n* = 497)	No	NA	No	No	No	No	No
Lepore 2012 [67]	PSA testing	Urban-NE (*n* = 490)	Yes	NA	No	Yes	Yes	No	No
Myers 2005 [68]	PSA testing	Urban-NE (*n* = 242)	Yes	NA	No	No	Yes	No	No
Myers 2011 [69]	PSA testing	Urban-NE (*n* = 313)	No	NA	No	No	Yes	No	No
Partin 2004 [70]	PSA testing	MW (*n* = 893)	Yes	NA	No	No	No	No	No
Rubel 2010 [71]	PSA testing	Urban-MW/NE/ South/West (*n* = 200)	No	NA	No	No	No	No	No
Schapira 2000 [72]	PSA testing	Urban-MW (*n* = 257)	No	NA	No	No	No	No	No^b^
Sheridan 2012 [73]	PSA testing	Urban-South (*n* = 128)	No	NA	No	No	Yes	No	No
Taylor 2013 [74]	PSA testing/DRE	Urban-South (*n* = 1,893)	No	NA	No	No	Yes	No	No
Volk 1999 [75, 76]	PSA testing	Urban-South (*n* = 158)	Yes	NA	No	No	Yes	Yes	No
Volk 2008 [77]	PSA testing	Urban-South (*n* = 450)	No	NA	Yes	No	Yes	No	Yes
Williams 2013 [78]	PSA testing	Urban-South (*n* = 543)	No	NA	Yes	No	Yes	No	No
Wolf 1996 [79]	PSA testing	Urban/Rural-NE/South (*n* = 205)	Yes	NA	Yes	No	Yes	No	No
PCa Treatment (*k* = 3)									
Barry 1997 [80]	Active surveillance v. prostatectomy v. medication	Urban-West (*n* = 227)	No	NA	No	No	No	No	No
Berry 2012 [81–83]	Active surveillance v. prostatectomy v. radiation	Urban-NE/South/West (*n* = 494)	No	NA	No	No	No	Yes	No
Marcus 2013 [51]	Active surveillance v. prostatectomy v. radiation	National (*n* = 208)	No	NA	No	No	Yes	No	No

Abbreviations: *US*, United States, *HS* high school diploma, *GED* general education development certificate, *NA* not applicable, *BCa* breast cancer, *BRCA1/BRCA2* breast cancer genes 1 and 2, *MW* Midwest, *NE* northeast, *BCT*, breast-conserving therapy (lumpectomy followed by radiation), *CRC* colorectal cancer, *FOBT* fecal occult blood testing, *FS* flexible sigmoidoscopy, *COL* colonoscopy, *DCBE* double contrast barium enema, *PSA* prostate-specific antigen, *DRE* digital rectal exam, *PCa* prostate cancer

^a^Did not directly assess health literacy, but argued for low literacy levels of the participants in discussion section of paper

^b^Used REALM to assess reading level. Over 80 % of participants had reading level about high school

### Inclusion of disadvantaged subgroups

**Fig. 2:**
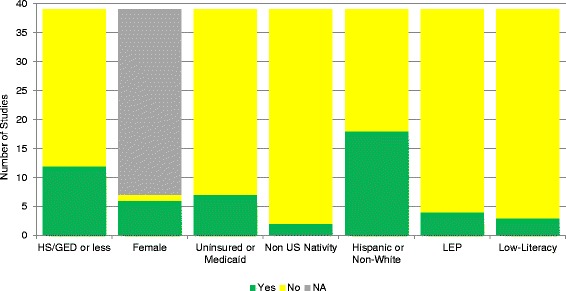
Frequency of Disadvantaged Subgroups Included in Randomized Trials of Cancer-Related Patient Decision Aids (*k* = 39 studies). Study samples met (Yes) or did not meet or did not report (No) criteria for being inclusive of specified subgroup, or criterion was not applicable (NA) to the cancer context (e.g., gender specific studies)

Twenty-three of 39 studies met one or more of our *a priori* criteria for inclusion of disadvantaged subgroups: HS or less education (*k* = 12); females (6-of-7 CRC screening studies); Medicaid or uninsured status (*k* = 7); non US nativity (*k* = 2); non-white race or Hispanic ethnicity (*k* = 18); LEP (*k* = 4); and low-literacy (*k* = 3), Fig. 2.

One-third of trials addressing decision making in BCa prevention or treatment targeted a socially disadvantaged group, but none of the three on BCa prevention in high risk groups included disadvantaged groups. One of five studies about BRCA genetic testing included representation of non-White patients (Black women). All six studies about CRC screening included at least one disadvantaged group; the sole study on CRC genetic testing did not. Most trials addressing PCa screening included at least one disadvantaged group, and one of these studies addressed five disadvantaged subgroups. Two of three PCa treatment trials included one disadvantaged group.

### Attention to SDH in PtDA development

We conducted a secondary search for additional qualitative and quantitative reports about the development of the PtDAs and identified clear evidence of attention to social disadvantage in the development of the PtDAs evaluated in 17 RCTs. This evidence (*k* = 12) plus the articles reporting the results of the included RCTs are referenced in Table 2. Nearly all of the developmental work used theoretical models to guide PtDA development, most frequently the Ottawa Decision Support Framework (ODSF). Several studies employed two or more PtDA formats and/or delivery modes, including coaching or counseling sessions, or utilized non-physician members of the health care team to deliver or assist with delivery of the PtDA. Racial/ethnic minorities and individuals with limited education and/or low health literacy were the subgroups most frequently targeted in PtDA development.

**Table 2:** Strategies used to develop, evaluate cancer-related PtDAs for disadvantaged social groups (*k* = 20)

Study	Eligibility criteria	Conceptual framework (if specified)	PtDA Format	Delivery Mode, setting	Sub-groups targeted in Dev.	Development (*k* = 17)	Evaluation (*k* = 14)
BCa, Genetic Testing							
Lerman 1997 [40]	Women 18-75y, ≥1 1^st^-degree relative with BCa and/or ovarian cancer	Behavioral decision-making models	Structured education (45-60 min) + semi-structured counseling (15-30 min)	By nurse or genetic counselor at oncology clinic	BLK	NR	Included PtDA-by- race interaction (no difference)
BCa, Treatment							
Jibaja-Weiss 2010 [50]	Women, early stage BCa	ODSF, EDAM	Interactive DVD/CD soap opera episodes, learning modules (43-207 min) [108]	By patient at clinic (with case manager assistance)	LOW-LIT, UNINS, BLK, HSP, SPN	Content developed by research team, expert advisory panel; usability, acceptability, demand tests conducted in subgroups [108]; adapted to SPN language [108]	*A priori* focus on multi-ethnic, low literacy women; use of low-literacy DCS
Marcus 2013 [51]	Women, early stage BCa	Social cognitive, self-regulation theories, Health Belief Model	Interactive, multimedia web-based program or CD (>60 min) + NCI print materials	By patient at home via computer or Internet	LOW-LIT	Content guided by formative research with patients and developed by NCI, literacy, patient education experts with 7^th^ grade readability target; usability, acceptability, demand tests conducted; reviewed for cultural appropriateness [51]; features of development sample NR	NR
Street 1995 [52]	Women, stage I/II BCa	NR	Interactive, multimedia program (30-45 min) vs. brochure (8 pages)	By patient at oncology clinic	LOW-ED	NR	Stratified/analyzed patients by age-education level (found older-less educated patients were less engaged)
CRC, Screening							
Miller 2011 [56]	50–74y, due for CRCS	Transtheoretical model	Interactive web-based program (~10.1 min)	By patient at primary care clinic	LOW-LIT, BLK	Content based on educational video [57] that was guided by subgroup interviews;[109, 110] navigation designed for “low-literacy” subgroup	Stratified/analyzed patients by literacy level (no difference); used REALM tool
Pignone 2000 [57]	50–75y, due for CRCS	Transtheoretical model	Educational video (11 min)	By patient at primary care clinic	LOW-ED, FEM, BLK	Content guided by subgroup interviews; assessed out-of-pocket cost concerns	NR
Ruffin 2007 [58]	50–70y, due for CRCS	Elaboration likelihood model	Interactive web-based education tool	By patient in community setting via Internet	FEM, BLK	Content and usability testing guided by subgroup FGs and interviews; race-, gender- and insurance-related issues, potential differences were explored [111].	*A priori* focus on blacks
Schroy 2011, 2012 [59, 60]	50–75y, due for CRCS	ODSF	Interactive DVD + personal risk assessment tool (20-30 min)	By patient at primary care clinic	LOW-ED, FEM, MCAID, BLK	Content guided by expert opinion, subgroup FGs and assessed out-of-pocket cost concerns, cultural sensitivity issues; usability tests conducted in subgroups	*A priori* focus on blacks and low-income non-Hispanic whites
PCa Screening							
Chan 2011 [63]	Men ≥40y, no PCa history	ODSF	Script, slides + video clips of role models (90-120 min), short booklets	By *promotores* to community center groups	LOW-ED, UNINS, MCAID, NONUS, HSP, SPN	Content guided by key informants, FGs; concept mapping conducted with men/spouses; adapted to SPN language	*A priori* focus on Hispanic and immigrant men
Lepore 2012 [67]	Men 45-70y, no PCa history	ODSF, social learning theory	Pamphlet + counseling sessions (~26.3 min)	By patient at home + health-educator via phone	LOW-ED, BLK	Content guided by expert opinion; feedback from men in target subgroups (including picture selection); cognitive interviews [112]	*A priori* focus on black and immigrant men; used low-literacy DCS
Myers 2005 [68]	Men 40-69y, no PCa or BPH history	PHM, AHP	Booklet + decision counseling session	By patient at home + health-educator via phone	LOW-ED, BLK	Content field-tested by literacy expert in face-to-face interviews with subgroup [113]; decision counseling protocol pilot tested in primary care practices in target community [113]	*A priori* focus on black men
Myers 2011 [69]	Men 50-69y, no PCa or BPH history	PHM, AHP	Booklet + decision counseling session	By patient at home + health-educator at primary care clinic	Refer to Myers 2005 [68]	Refer to Myers 2005 [68]	NR
Sheridan 2012 [73]	Men 40-80y, no PCa history	NR	Video (12 min) + coaching session (8 min)	By patient at primary care clinic (with research assistance)	NONWHT	Content pre-tested in FGs, cognitive interviews, usability tests in subgroups	NR
Taylor 2013 [74]	Men 45-70y, no PCa history	NR	Interactive web-based tool + booklet (~34 min)	By patients at home	LOW-LIT, BLK	Content guided by expert opinion, key informants, subgroup FGs [114, 115]; adapted to 8^th^ grade readability by plain language expert; usability, acceptability, demand tests conducted in subgroups with limited computer/internet skills [114, 116]	NR
Volk 2003 [76]	Men 45-70y, no PCa history	NR	Videotape (~20 min) + brochure	By patient at PC clinic	NR	NR	Stratified analysis by race (found difference in screening rates)
Volk 2008 [77]	Men 40-70y, no PCa history	ODSF, EDAM	Interactive DVD/CD-based soap opera episodes, learning modules (53-68 min)	By patient at public hospital clinic (with research assistance)	LOW-LIT, UNINS, BLK	Content tailored using racial/ethnic concordance, social matching; acceptability tests conducted in subgroups	Stratified analysis by literacy; *a priori* interest in low-literacy black men (found differences in acceptability, decisional conflict); used low-literacy DCS
Williams 2013 [78]	Men 40-70y, no PCa history	NR	Booklet	By patient at home *vs.* patient at cancer screening clinic	LOW-LIT, BLK	Content by expert opinion, key informants, subgroup FGs [114, 115]; adapted to 8^th^ grade readability by plain language expert; usability tests conducted in subgroups [114]	Stratified sub-analyses by race, including race-by delivery mode interaction (no differences)
Wolf 1996 [79]	Men ≥50y, no PSA testing or PCa history	NR	Script about PSA screening	By research assistant at PC clinic	LOW-ED, NONWHT	Content developed by physician experts; piloted via subgroup interviews; assessed for comprehensibility	Stratified sub-analyses by income, education (no difference)
PCa Treatment							
Berry 2013 [81–83]	Men, localized PCa	ODSF	Interactive web-based program (~46 min) + usual patient education	Patient at home or oncology clinic	BLK	Content tailored by race via subgroup FGs, interviews [117, 118]; feasibility, acceptability tests conducted	Efficacy evaluation discussed decisional conflict by race (found differences in decisional conflict, perceived decision support)
Marcus 2013 [51]	Men, newly diagnosed localized PCa	Social cognitive, self-regulation theories, Health Belief Model	Interactive, multimedia web-based or CD program (>60 min) + NCI print materials	By patient at home via computer/internet	LOW-LIT, BLK	Content guided by formative research with patients and developed by NCI, literacy/patient education experts with 7^th^ grade readability target; usability, acceptability, demand tests conducted; reviewed for cultural appropriateness [51]; features of development sample NR [119]	NR

*DM* decision-making, *BCa* breast cancer, *ODSF* Ottawa Decision Support Framework, *EDAM* edutainment decision aid model, *IDM* informed decision-making, LIT low literacy, *UNINS* uninsured, *BLK* black, *HSP* Hispanic, *SPN* Spanish language, *DCS* decisional conflict scale, *SDM* shared decision-making, *NCI* National Cancer Institute, NR not reported, *CRCS* colorectal cancer screening, *REALM* Rapid Estimate of Adult Literacy in Medicine, *FEM* female, *FGs* focus groups, *MCAID* Medicaid, *PCa* prostate cancer, *NONUS* non US-born, *PSA* prostate-specific antigen, *NONWHT* non-white

Development strategies included focus groups and/or key informant interviews, cognitive interviews or questionnaires to guide content modification, concept mapping, and expert assessments of plain language readability and/or cultural appropriateness. Several PtDAs featured characters and/or narrators similar to the race/ethnicity or social status of target subgroups. Two studies adapted the PtDA to the users’ preferred language (Spanish). The development process for several of the PtDAs employed usability and/or feasibility testing, such as acceptability or comprehensibility assessments, to explore and modify design features (e.g., language, narration, video, animation, touch screen formats) that would make the material more accessible for participants with limited health literacy, numeracy or computer/internet skills [50, 51, 56, 58–60, 73, 74, 77, 78, 81–83]. As part of the development process, several studies conducted activities associated with tracking estimated and/or actual use of the PtDA (demand testing). The average time patients spent using the PtDAs ranged from 10 to 207 min.

### Attention to SDH in PtDA evaluation

We identified 14 studies with clear evidence of attention to social disadvantage in evaluating the PtDA, according to our stated criteria (Table 2). Most were included based on the authors’ *a priori* objectives to evaluate the effectiveness of PtDAs in specific populations (e.g., Black men, Hispanic men, low-literacy populations), but we also found articles that stratified analyses by or discussed low versus high education/income, low versus adequate literacy or race. Three studies also utilized a low-literacy version of O’Connor’s Decisional Conflict Scale (DCS) to reduce measurement error in limited literacy populations. Although several studies classified participants as “low-literacy”, only one study measured literacy using standard criteria (the Rapid Estimate of Adult Literacy in Medicine, REALM).

## Discussion

This review assessed the extent to which disadvantaged subgroups have been included in US RCTs of cancer-related PtDAs and summarized the strategies employed in developing and evaluating PtDAs that address one or more SDH. Our findings revealed that nearly 60 % of studies were inclusive of disadvantaged participants based on at least one criterion associated with health inequities [36], most frequently gender, race/ethnicity (required reporting statistics for research projects funded by the US government), and education. Inclusion of subgroups disadvantaged by their insurance status, LEP, low-literacy or non US-nativity was far less common. The processes used to develop the PtDAs, as well as the features of the tools that promoted or hindered decision making in disadvantaged subgroups, were not well-described in most studies. Our purpose was to glean key lessons from those studies in which SDH were prominent in the development or evaluation of cancer-related PtDAs and to highlight the need for more attention to the comprehensive range of social factors that may influence decision making among disadvantaged patients. Below, we outline our observations and present opportunities for future research.

### Inclusion of disadvantaged subgroups

Although diversity in the US is represented in a majority of the studies reviewed, disadvantaged subgroups were not included in some PtDA trials in numbers proportional to their burden of disease for the decision making contexts being targeted. A key example is our finding that disadvantaged subgroups were not included in most studies that focused on BRCA genetic testing or BCa prevention in high risk populations. These represent important targets for PtDA studies given current research that demonstrates significant disparities experienced by Black and Hispanic women in awareness and use of BRCA testing/genetic counseling [84–87]. Another important example is our finding that few cancer treatment trials were inclusive of Black men and women, despite their disproportionately high rates of incidence and mortality for PCa or mortality for BCa, respectively [4].

These findings stimulate important questions about how to establish an inventory of PtDAs that is most relevant to disadvantaged social groups and their clinicians. For example, we identified no PtDAs evaluated in US RCTs that addressed the decision to give the hotly debated human papillomavirus (HPV) vaccination [88, 89] to adolescents and young women to prevent cervical cancer. Cervical cancer morbidity and mortality rates are highest among Hispanic, Black, American Indian and Alaskan Native women in the US [4], while disparities in awareness of and knowledge about HPV persist among these populations [90]. Lung cancer morbidity and mortality are highest among Black men in the US [4], and lung cancer screening, in combination with smoking cessation interventions, is now recommended for high risk populations [91]. PtDAs to support prevention and screening decisions for these conditions may be useful to disadvantaged populations. PtDAs for cancer surveillance among disadvantaged populations is also an important but understudied area. One study of Hispanic and immigrant men found that not all patients fully recognized that watchful waiting, or opting not to be screened, can also be responsible choices related to PCa [63]. Other racial/ethnic differences in treatments discussed, preferred and received for localized PCa (surgery, radiation therapy, and active surveillance) have also been reported [92].

### Attention to SDH in PtDA development and evaluation

#### Education and health literacy

Education and health literacy were among the most widely addressed disadvantages in the studies reviewed. Most studies that focused on addressing limited education in PtDA development targeted 7^th^ to 8^th^ grade readability as the threshold for ease-of-use. The studies that identified low-literacy patients as an intended audience for the PtDA did not consistently report how this construct was defined and/or measured. Focus group and individual interviews were frequently used to determine whether the PtDAs were accessible to limited education/low literacy patients. Myers et al., for example, engaged a literacy expert from a community-based health promotion organization in Philadelphia to facilitate this process. The expert conducted face-to-face interviews to ascertain whether Black men recognized the purpose of a PCa screening information booklet and understood its related language, terms and concepts [68]. Through this process, the authors learned that the medical terms should be simplified and more pictures should be included. They found that an increased emphasis on the issue of PCa screening as a decision to be made in partnership with a physician was also needed – many men thought the purpose of the PtDA was to *promote* PCa screening rather than to support decision making.

Several studies found that the acceptability and demand for PtDAs varied between advantaged and disadvantaged subgroups based on PtDA format and other design attributes. Volk et al*.* found that low compared to high literacy users were willing to spend more time viewing the PtDA [77]. Marcus et al. [51] and Taylor et al. [74] found that participants were more likely to use print-based versus web-based PtDAs (web-based PtDA use was highest among White participants who reported frequent internet use overall) [74]. Other studies acknowledged that disadvantaged patients needed more help utilizing PtDAs and facilitated delivery of the intervention using non-physician members of the healthcare team. Like the use of patient navigators to provide psychosocial and logistical support for disadvantaged cancer patients [93, 94], the use of health coaches to guide decision making [95] for disadvantaged populations may also be needed. Appropriate consideration should be given to both PtDA design and the other factors that may influence disadvantaged participants’ exposure to the intervention. By engaging patients early in the development process, the most appropriate PtDA formats and delivery strategies can be identified.

The influence of PtDAs on facilitating SDM via improved patient-provider communication, or even patients’ interest in participating in SDM, was discussed rarely in identified studies. By definition, being health literate includes being able to act on health information [96]. Several factors may influence whether patients engage in SDM with their physicians (some are highlighted below as part of our discussion regarding social norms and attitudes). Although LEP, the use of translators in explaining treatment options, and the lack of available educational materials in a patient’s preferred language, for example, are factors known to influence patients’ reliance on physician recommendations over more active participation in decision making [29], PtDAs designed for disadvantaged populations may play a role in improving these aspects of patient-provider interactions. More information is needed to better understand this issue, particularly in light of a recent systematic review finding that SDM interventions were more beneficial to disadvantaged, compared to advantaged subgroups [97].

Another notable finding is the limited consideration of numeracy in relation to health literacy. Two studies described addressing numeracy as part of the PtDA development process [59, 83]; one study measured and reported participants’ baseline numeracy skills [74]. As reported in a recent study [98], numeracy and health literacy have largely been treated as separate concepts in the literature despite indications that PtDAs designed for patients with different levels of health literacy may not support the needs of patients with disparate numeracy skills. This is another important area for future research.

#### Race, ethnicity and related social norms and attitudes

Matching the race/ethnicity of the actors/models to that of the intended audience is a commonly used strategy in tailoring PtDAs for disadvantaged social groups. Jibaja-Weiss et al. produced six versions of PtDA soap opera segments so that users were able to receive information about BCa treatment from female characters similar to them in race/ethnicity, preferred language and age [50]. Volk and colleagues employed a multiethnic, blue-collar cast and tailored the main character to the viewer through a series of questions completed upon entering the program [77]. The program included a values clarification, social-matching exercise that asked the viewer to “pick who is most like you” with regard to his feelings about PCa screening. The strategy of promoting racial/ethnic concordance to improve processes of care and outcomes for minorities has been extensively studied, with mixed results [99, 100]. The underlying assumption is that people are able to better identify with others who look like them or share similar language or culture. In turn, this may improve their interactions (e.g., greater satisfaction, trust, willingness to ask questions on the part of patients; less uncertainty, bias, stereotyping on the part of providers; better patient-provider communication overall). However, the influence of patient-provider race-concordance on health care interactions varies within and across race/ethnicity based on related SDH (e.g., income, citizenship status, language) [99–101]. These differences may be present in other norms and attitudes related to race/ethnicity and culture (e.g. attitudes about surgery; opinions of spouse, family and friends; concerns about body image) [102]. Reflecting the racial/ethnic diversity of target audiences in PtDAs is most certainly an important patient engagement strategy. However, it is also important to recognize that heterogeneity exists within racial/ethnic groups. More research is needed to better understand whether PtDAs can help to improve decisional outcomes that may be influenced by race/ethnicity and related social norms and attitudes.

#### Economic instability

Although few studies indicated that patients were asked about economic issues during PtDA development, the need to disentangle the various ways in which economic instability may influence patient decision making is an important area for future research. As noted, Jibaja-Weiss et al. [50] found that PtDA exposure was associated with increased uptake of mastectomy, rather than breast conserving therapy, among low-literacy Black and Hispanic women with early stage BCa. This finding differed from published studies linking PtDAs with the choice to undergo more conservative surgical options [20, 103]. The reason for this difference remains unclear, but other studies of low income women in the US suggest that economic factors (e.g. uninsured or Medicaid status, insurance co-payments, out-of-pocket expenses, concerns about missed work) influence their treatment choices [29, 104, 105].

#### Other SDH

Multiple factors affect the health and health care received by disadvantaged populations, including many SDH that were not addressed in this research. In some decision contexts, for example, it may be important to use PtDAs to help patients consider how attributes of their neighborhoods and built environments (e.g., housing, transportation, public safety, other public services, environmental noise and pollutants) figure into their decision making. We do not mean to suggest that all SDH can be practically addressed in every PtDA for disadvantaged patients. However, if the purpose of PtDAs is to support high quality SDM in the cancer care continuum, we believe that addressing only education and/or health literacy when developing tools for patients who face multiple social disadvantages falls short of that goal.

### Limitations and strengths

This review has several potential limitations. First, despite our efforts to identify all US RCTs of cancer-related PtDAs, it is possible that our search missed relevant RCTs that fit our inclusion criteria. We attempted to minimize this possibility by comparing our list of eligible studies with those in recently published systematic reviews of PtDAs using similar search and eligibility criteria [20, 106, 107]. By limiting our review to RCTs, we may have missed studies that were more inclusive of disadvantaged subgroups or that employed PtDA development and evaluation strategies other than those described in the articles identified for this review. Yet, given the large number of RCTs of cancer-related PtDAs that have been conducted [20], and given the importance of the Cochrane review, limiting this review to the Cochrane criteria of RCTs seems appropriate. We did include citing and cited publications related to the RCTs that cast a larger net for information on PtDA development. We did not, however, contact the study authors for additional information, which may have potentially caused us to miss relevant information not consistently reported. We attempted to address this problem by focusing our data collection on socioeconomic and cultural constructs most widely and consistently reported in the literature. We acknowledge that other constructs related to social disadvantage, such as social capital, place of residence, physical and mental disability, sexual orientation or religion, are important characteristics that should be more consistently reported and evaluated in future PtDA research. We were unable to identify a cancer-related PtDA trial that addressed social disadvantages related to these characteristics. Limitations present in all systematic reviews, including publication bias and potential researcher bias in establishing study inclusion criteria, may also have been present in this study.

## Conclusions

The number of cancer-related PtDAs will continue to proliferate in the coming years as such tools are advocated as a strategy to facilitate patient engagement in SDM. While it may be tempting to evaluate or utilize PtDAs developed without attention to SDH and argue that the tools are effective based on improvements in knowledge measured broadly or other decisional outcomes, we believe such an approach misses many important considerations in providing decision support that may ultimately undermine quality decision-making processes. This review addresses a gap in the literature by summarizing the extent to which cancer-related PtDAs have been used by disadvantaged patients in the US and by highlighting the strategies and lessons learned in the development and evaluation of the handful of PtDAs specifically tailored for disadvantaged social groups. Such information may be used to guide clinicians as they attempt to identify among a large and expanding inventory of available PtDAs those tools that that were designed to support the decision making needs of their socially disadvantaged patients. It may also be used to inform the development of new or complementary interventions to support high quality SDM processes among disadvantaged populations.

## Abbreviations

BCa, breast cancer; BCT, breast-conserving therapy; BRCA1/BRCA2, breast cancer genes 1 and 2; COL, colonoscopy; CRC, colorectal cancer; DCBE, double contrast barium enema; DCS, decisional conflict scale; DRE, digital rectal exam; FOBT, fecal occult blood testing; FS, flexible sigmoidoscopy; GED, general education development certificate; HS, high school diploma; HPV, human papillomavirus; LEP, limited English proficiency; MSI, microsatellite instability; MW, Midwest; NA, not applicable; NE, northeast; ODSF, Ottawa Decision Support Framework; PCa, prostate cancer; PSA, prostate-specific antigen; PtDA, patient decision aid; RCT, randomized controlled trial; REALM, rapid estimate of adult literacy in medicine; SDM, shared decision making; SDH, social determinants of health; US, United States.

## Additional file

Additional file 1: Electronic search strategy used for PubMed. (PDF 8 kb)
