# Supplementary material for: Are cancer-related decision aids appropriate for socially disadvantaged patients? A systematic review of US randomized controlled trials
Source: BMC Med Inform Decis Mak. 2016 Jun 6;16:64. doi: 10.1186/s12911-016-0303-6 (PMC4896023; doi:10.1186/s12911-016-0303-6)
Supplement: Additional file 1: — Electronic search strategy used for PubMed. (PDF 8 kb) [file 12911_2016_303_MOESM1_ESM.pdf]

Additional File 1. Electronic Search Strategy Used for PubMed

#3	#1 AND #2
#2	("2010/01/01"[PDAT] : "2013/12/31"[PDAT])
#1	("decision aid"[All Fields] OR "patient decision making"[All Fields] OR "patient decision-making"[All Fields] OR "shared decision making"[All Fields] OR "shared decision-making"[All Fields] OR "informed decision making"[All Fields] OR "informed decision-making"[All Fields] OR "informed choice"[All Fields]) AND ("randomized controlled"[All Fields] OR "RCT"[All Fields] OR "clinical trial"[All Fields] OR "randomized trial"[All Fields]) AND ("neoplasms"[MeSH Terms] OR "neoplasms"[All Fields] OR "cancer"[All Fields])
